# Death from Gall-Stone Colic

**Published:** 1911-06-03

**Authors:** 


### Medicine.

### DEATH FROM GALL-STONE COLIC.

Gall-stones may lead to death directly as the result of the acute colic they may produce, the patient at the same time suffering from a fatty heart as part of the general obesity that is so frequently associated with the development of gall-stones. The intensity of the pain in these cases may be sufficient to cause fatal failure of the fatty heart, as in the following instance:—

A married woman, fifty-three years of age, and now enormously fat, had had eleven children and seven miscarriages. The only previous illnesses of importance that she gave a history of were scarlet fever when she was eleven years of age, and an attack of acute abdominal pain, similar to that from which she was now suffering, but much milder in degree, twenty-five years ago. There may have been minor attacks of pain in the interval between then and now, but they had not been particularly severe. For the last two months the patient had been feeling unwell with symptoms of flatulence and dyspepsia, but she was not seriously ill until seized on March 2 with very violent pain in the upper part of the abdomen, the latter continuing intermittently until she was admitted to the hospital on March 8. During the week preceding her admission she had been sick many times, but her bowels had been opened regularly. She thought that the pains were worst about thirty minutes after food, but it was difficult to make out anything very definite upon examination because she was so exceedingly stout. The skin and sclerotics were of a slightly yellowish tint suggestive of jaundice, though they were not deeply tinged. From her general appearance one gathered that she was in great pain, and on palpation the abdomen was tender, especially above the umbilicus and to the right of it; there was no rigidity of the abdominal wall and it moved fairly well with respiration. The lungs were emphysematous and non-consonating râles and rhonchi were heard over both sides of the chest. The heart was difficult to hear, partly on account of the emphysema of the lungs, partly on account of the sounds produced by the bronchitis, partly on account of the general fatness of the subcutaneous tissue, and partly on account of the feeble action of the heart itself; but no bruit could be heard and there was no œdema of the legs, though the urine contained a trace of albumin.

By March 15 the patient was no longer in pain, but she still suffered from bronchitis and was very short of breath. On March 19 the yellowish colour of the skin became more pronounced and the acute pain in the right upper part of the abdomen recurred with greater intensity and the patient was repeatedly sick. Morphia injections were required on account of the great severity of the colic. About 4 p.m. upon March 21 the general condition of the patient became suddenly worse; the respirations being rapid and shallow, the pulse exceedingly feeble, and the face and extremities cyanosed. Consciousness was retained until a few minutes before death, which occurred at ten minutes past four.

Previous to post-mortem examination the most obvious feature in the case was the acute failure of the heart and there was some question as to whether the pains in the upper part of the abdomen may not have been referred in some way from the heart; at the autopsy, however, a large solitary gall-stone rather bigger than an ordinary corn was found impacted in the common bile-duct close to the ampulla of Vater, where it was beginning to press through the mucous membrane into the duodenum in such a way as to suggest that had the patient survived a few days longer the calculus would have passed into the bowel with complete relief to the abdominal symptoms. The gall-bladder was contracted, small and embedded in adhesions, the result of former local peritonitis, due no doubt to the inflammation associated with the gall-stone. It seems likely that this big stone had begun to pass down the cystic duct about March 2 and that it had reached the common bile-duct about March 8; it then ceased to advance for a few days, without yet being entirely impacted in the common bile-duct; that on March 19 the stone began to pass down the common bile-duct again, jaundice now beginning to deepen once more and the pains recurring with increased intensity; the stone had very nearly succeeded in passing the whole way through when the patient died of acute failure of the heart. The latter weighed 548 grams; but the greater part of this increase in weight was due to fat and not to heart muscle. Indeed, the actual thickness of the myocardium was less than normal, and even that which looked like heart muscle was markedly streaked with yellow lines of bile-stained fat, whilst over the whole surface of the heart there was such an excess of adipose tissue that it was not at all surprising that heart failure came on acutely at the end. Nevertheless up till the time of the acute biliary colic the heart was well able to do such work as was required of it, and the final factor in producing the fatal cardiac failure was a recrudescence of severe biliary colic, which, therefore, was the immediate cause of death.

